# Two classes of EF1-family translational GTPases encoded by giant viruses

**DOI:** 10.1093/nar/gkz296

**Published:** 2019-04-24

**Authors:** Alexandra Zinoviev, Kazushige Kuroha, Tatyana V Pestova, Christopher U T Hellen

**Affiliations:** Department of Cell Biology, SUNY Downstate Medical Center, 450 Clarkson Avenue, MSC 44, Brooklyn, NY 11203, USA

## Abstract

Giant viruses have extraordinarily large dsDNA genomes, and exceptionally, they encode various components of the translation apparatus, including tRNAs, aminoacyl-tRNA synthetases and translation factors. Here, we focused on the elongation factor 1 (EF1) family of viral translational GTPases (trGTPases), using computational and functional approaches to shed light on their functions. Multiple sequence alignment indicated that these trGTPases clustered into two groups epitomized by members of *Mimiviridae* and *Marseilleviridae*, respectively. trGTPases in the first group were more closely related to GTP-binding protein 1 (GTPBP1), whereas trGTPases in the second group were closer to eEF1A, eRF3 and Hbs1. Functional characterization of representative GTPBP1-like trGTPases (encoded by Hirudovirus, Catovirus and Moumouvirus) using *in vitro* reconstitution revealed that they possess eEF1A-like activity and can deliver cognate aa-tRNAs to the ribosomal A site during translation elongation. By contrast, representative eEF1A/eRF3/Hbs1-like viral trGTPases, encoded by Marseillevirus and Lausannevirus, have eRF3-like termination activity and stimulate peptide release by eRF1. Our analysis identified specific aspects of the functioning of these viral trGTPases with eRF1 of human, amoebal and Marseillevirus origin.

## INTRODUCTION

The eukaryotic Nucleocytoplasmic Large DNA Viruses (NCLDV) form the proposed order *Megavirales*, which includes the *Ascoviridae, Asfarviridae, Iridoviridae, Marseilleviridae, Mimiviridae, Phycodnaviridae, Poxviridae* and the proposed *Pandoraviridae* and *Pithoviridae* families (1–3). Several of these groups contain giant viruses, which have extraordinarily large dsDNA genomes (0.5–2.5 Mb) and virus particles, and infect a wide phylogenetic range of eukaryotes, including amoebae, phytoplankton and microzooplankton (4–7). The first giant virus to be described, *Acanthamoeba polyphaga* mimivirus (APMV), was discovered in amoeba from water in a hospital cooling tower during studies of a pneumonia outbreak (8), and many others have subsequently been identified (4,6,7,9–12). An exceptional property of these viruses is that they encode components of the protein synthesis apparatus, including tRNAs, aminoacyl-tRNA synthetases and translation factors (6,10,11,13–17). Prior to the discovery of APMV, the presence of genes encoding multiple components of the translation apparatus had been thought to be restricted to cellular organisms.

The evolutionary histories of these genes suggest that they had multiple origins, and in different viruses were acquired through convergent capture of host genes from diverse hosts and by inter-virus exchange, followed by gradual loss (3,6,18). These observations suggest that there are circumstances in which these viruses benefit from the acquisition and retention of translation-related genes. Importantly, viral genes that encode the translational components are transcribed in virus-infected cells (19–22; http://www.igs.cnrs-mrs.fr/mimivirus/), and in one instance (APMV initiation factor (eIF) 4A), one of these gene products has been found to influence viral mRNA translation, growth and fitness (21). However, except for two APMV aminoacyl-tRNA synthetases, the biochemical activity of giant virus-encoded components of the translation apparatus has not been confirmed (23).

Here we focused on one of the virally encoded protein synthesis-related gene groups: translational GTPases (trGTPases). TrGTPases are a superfamily of proteins that belong to the P-loop GTPase superclass and participate in all stages of translation. They contain a highly conserved GTPase (G) domain followed by two β-barrel domains, and can be divided into EF1, SelB, EF2 and IF2 families (24,25). Different giant viruses encode different complements of trGTPases, including eIF2γ (a member of SelB family), eIF5B (a member of IF2 family) and members of the EF1 family. The EF1 family is epitomized by bacterial and eukaryotic elongation factors EF-Tu and eEF1A, the eukaryotic release factor eRF3, and the mRNA surveillance factor Hbs1. eEF1A, eRF3 and Hbs1 are also closely related to eukaryotic GTPBPs, which constitute a relatively divergent group in the SelB family (25). The members of EF1 family and GTPBP1 have a similar function of delivering their binding partners to the ribosomal A site in a GTP-dependent manner. Thus, EF-Tu and eEF1A deliver aa-tRNA to the A site during translation elongation (26). eRF3 forms a ternary complex with GTP and eRF1, delivering eRF1 to the A site of pre-termination complexes. eRF1 and eRF3 function interdependently, with eRF1 promoting eRF3′s GTP-binding and ribosome-dependent GTPase activities (27–30) and eRF3 enhancing eRF1-mediated peptide release (31). Hbs1, which is involved in ‘no-go’ and ‘non-stop’ mRNA decay, delivers to the A site a paralogue of eRF1 called Pelota, which participates in splitting of stalled 80S ribosomes (32,33). A recent study (34) revealed that GTPBP1 also possesses eEF1A-like elongation activity, delivering aa-tRNA to the A site, but the kinetics of GTPBP1-mediated elongation argues against its functioning in elongation, but supports involvement in mRNA surveillance (34,35). In each case, the GTP is hydrolyzed after binding of the ternary complex to the ribosome, which reduces the affinity of the trGTPase to its partner, leading to its release into the A site and to dissociation of the trGTPase (26,36–38).

EF1 family members encoded by giant viruses have variously been classified as GTPBP1 (13), EF-Tu (16), eEF1A (3,10,17) and as a GTP-binding translation elongation/initiation factor (11). In this study, we applied computational and functional *in vitro* reconstitution approaches to clarify our understanding of this group of giant virus translation factors. We found that they segregate into two classes epitomized by members of *Mimiviridae* and *Marseilleviridae*, respectively. Analysis of GTPases encoded by representative members of these families revealed that they possess eEF1A-like elongation and eRF3-like termination activities, respectively.

## MATERIALS AND METHODS

### Plasmids

The following expression vectors have been described: His_6_-tagged human eIF1 and eIF1A (39), eIF4A and eIF4B (40), eIF4GI_736–1115_ (eIF4Gm) (41), eIF5 (42), eRF1 and eRF3aC lacking the N-terminal 138 a.a. (referred to as eRF3 in the text) (43,44), Pelota and Hbs1 (32) and His_6_-tagged *Escherichia coli* methionyl tRNA synthetase (45).

Vectors for expression of N-terminally His_6_-tagged viral proteins were prepared by inserting the relevant ORFs into pET28a using the indicated restriction sites to generate pET28a-CTV1-GTPase (Nde1/BamHI), pET28a-HV-Sang-GTPase (Nhe1/BamHI), pET28a-MVMV-GTPase (Nhe1/BamHI), pET28a-Ac-eRF1 (Nhe1/BamHI), pET28a-MVMV-eRF1 (Nhe1/BamHI), pET28a-MoV-mon-GTPase (BamHI/XhoI) and pET28a-LV-GTPase (BamHI/NotI) (GeneWiz, South Plainfield, NJ, USA). The vector for expression of eRF1_Δmini-domain_ (in which a.a. 328–373 has been substituted by the sequence ASTAAS) was generated by mutagenesis of the *wt* eRF1 vector (NorClone Biotech Laboratories, London, Ontario, Canada).

The following transcription vectors have been described: tRNA_i_^Met^ (46), tRNA^Leu^-AAG (47) and MVHL-STOP, MF-STOP and MLHL-STOP mRNAs (31,34). The MFFF-STOP mRNA transcription vector was made similarly by inserting an appropriate DNA fragment flanked by a T7 promoter and a HindIII restriction site into pUC57 (GeneWiz).

### Purification of mammalian and viral translation factors and ribosomal subunits

Native 40S and 60S ribosomal subunits, eIF2, eIF3, eIF5B, eEF1H, eEF2 and total native aminoacyl-tRNA synthetases were purified from rabbit reticulocyte lysate (Green Hectares, Oregon, WI, USA) as described (48,49). Recombinant His_6_-tagged eIF1, eIF1A, eIF4A, eIF4B, eIF4G_736–1115_, eIF5, eRF1, eRF3, Pelota, Hbs1, GTPBP1 and *Escherichia coli* methionyl tRNA synthetase were expressed in *E. coli* BL21 (DE3) and purified as described ((31) and references therein; 32,49). eRF1_Δmini-domain_ was purified in a similar way to the *wt* eRF1 (43).

Recombinant His_6_-tagged MoV-mon GTPase, LV GTPase, CTV1 GTPase, HV-Sang GTPase, MVMV GTPase, Ac eRF1 and MVMV eRF1 were expressed in 4 l of *E. coli* BL21 DE3 Star (Invitrogen). Protein production was induced by addition of 0.5 mM IPTG, after which cells were grown for 16 h at 16°C or for 2 h at 16°C for MVMV eRF1. All proteins were isolated by affinity chromatography on Ni-NTA agarose followed by FPLC on a MonoS 5/50 GL column for all GTPases and on a MonoQ 5/50 GL column for all eRF1s.

### Preparation of mRNAs and tRNAs

tRNA_i_^Met^, tRNA^Leu^-AAG and all mRNAs were *in vitro* transcribed using T7 RNA polymerase. Native calf liver total tRNA was from Promega. Yeast native tRNA^Phe^ was purified as described (50). *In vitro* transcribed tRNA_i_^Met^ was aminoacylated using *E. coli* methionyl tRNA synthetase (49). Elongator tRNAs were aminoacylated using total native aminoacyl-tRNA synthetases or purified specific yeast tRNA synthetases (49,51). Leu-tRNA^Leu^ and Phe-tRNA^Phe^ were separated from non-aminoacylated tRNAs by HPLC on an RP18 column. Native total aa-tRNAs were purified as described (52).

### Assembly and toe-printing analysis of ribosomal complexes

Ribosomal complexes were assembled essentially as described (31,49,53). First, 48S initiation complexes were formed by incubating 25 nM mRNA with 60 nM 40S subunits, 350 nM eIF1, 350 nM eIF1A, 90 nM eIF2, 60 nM eIF3, 300 nM eIF4A, 60 nM eIF4B, 250 nM eIF4G_736–1115_ and 100 nM Met-tRNA_i_^Met^ in 400 μl of buffer A (20 mM Tris–HCl pH 7.5, 3.8 mM MgCl_2_, 100 mM KCl, 0.25 mM spermidine, 2 mM DTT) supplemented with 1 mM ATP, 0.3 mM GTP and 1 U/μl RiboLock RNase inhibitor (Thermo Scientific) for 10 min at 37°C. To obtain 80S ICs, reaction mixtures were supplemented with 90 nM of 60S subunits, 200 nM eIF5 and 60 nM eIF5B, and incubation continued for an additional 10 min. To obtain pre-TCs, elongation was carried out by mixing 80S IC reactions with 60 nM eEF2, 150 nM eEF1H and 1 μM native total calf liver aa-tRNA, after which incubation continued at 37°C for an additional 15 min. Assembled 80S ICs and pre-TCs were purified by centrifugation in a Beckman SW55 rotor for 1 h 35 min at 4°C and 53 000 rpm in 10–30% linear sucrose density gradients (SDG) prepared in buffer B (20 mM Tris–HCl, pH 7.5, 2.5 mM MgCl_2_, 100 mM KCl, 0.25 mM spermidine, 2 mM DTT), and stored at –80°C.

Elongation experiments were carried out by mixing SDG-purified 80S ICs with 60 nM eEF2, 300 nM individual aa-tRNAs and 150 nM of eEF1H, HV-Sang GTPase, CTV1 GTPase, MoV-mon GTPases, LV GTPase or MVMV GTPase, after which incubation continued at 37°C for an additional 15 min (unless otherwise indicated). In time course experiments (Figure 2E), the elongation reactions were stopped by elevating the Mg^2+^ concentration to 20 mM before initiating primer extension. Resulting ribosomal complexes were analyzed by toe-printing (49) using AMV reverse transcriptase (Promega) and [^32^P]-labeled oligonucleotide primers complementary to nt 149–166 of MLHL-STOP and MFFF-STOP mRNAs, nt 143–160 of MF-STOP mRNA, or nt 209–227 of MVHL-STOP mRNA. cDNA products were resolved in 6% polyacrylamide sequencing gels followed by autoradiography.

Termination experiments were carried out by mixing 3.75 nM of SDG-purified pre-TCs with various combinations of 50 nM MoV-mon GTPase, 50 nM LV GTPase, 50 nM CTV1 GTPase, 50 nM HV-Sang GTPase, 50 nM MVMV GTPase, 50 nM human eRF3, 30 nM human eRF1, 30 nM human eRF1_Δmini-domain_, 30 nM Ac eRF1 and 30 nM MVMV eRF1 and incubating the reaction mixtures at 37°C for 15 min. The resulting ribosomal complexes were also analyzed by toe-printing, as described above.

### UV crosslinking assay

0.3–0.5 μM MoV-mon GTPase, LV GTPase, CTV1 GTPase, or HV-Sang GTPase were incubated for 5 min at 30°C in 30 μl buffer B with 120 nM [α-^32^P]GTP individually or in combination with 0.4 μM deacylated native yeast tRNA^Phe^ or amino-acylated native yeast Phe-tRNA^Phe^, in the presence of 0.5 mg/ml casein. Assembled complexes were irradiated on ice for 10 min at 254 nm, treated with RNAses A and T1 for 15 min at 37°C, and analyzed by 4–12% SDS-PAGE followed by fluorescent SYPRO staining (Invitrogen) (to ensure equal loading) and autoradiography.

### NTP hydrolysis assays

0.25 μM MoV-mon GTPase, LV GTPase, CTV1 GTPase, HV-Sang GTPase, MVMV GTPase, eRF3 or Hbs1 were incubated for 20 min at 37°C in 10 μl buffer B with 40 nM [α-^32^P]GTP in the presence/absence of different combinations of 0.1 μM 80S ribosomes, 10 nM SDG-purified 80S Elongation complexes (containing UUC codon in the A site), 0.5 μM Phe-tRNA^Phe^, 0.25 μM Pelota, 0.25 μM eRF1, 0.25 μM eRF1_Δmini-domain_, 0.25 μM Ac eRF1, and 0.25 μM MVMV eRF1. [α-^32^P]GTP and [α-^32^P]GDP in the reaction mixtures were separated using TLC on polyethyleneimine cellulose by spotting 1.5 μl aliquots onto the plates for chromatography done using 0.8 M LiCl/0.8 M acetic acid. In Figures 3A, 5B and Supplementary Figure S1C, the efficiency of GTP hydrolysis was calculated by scanning the TLC plates using an Amersham Typhoon (GE) and quantifying the images using ImageQuantTL software. In Supplementary Figure S2A, a similar experiment was done where [α-^32^P]GTP was replaced by [α-^32^P]UTP or [γ-^32^P]ATP, and the TLC separated [α-^32^P]UTP and [α-^32^P]UDP or [γ-^32^P]ATP and [^32^P]P_i_, respectively.

### Peptide release assay

For peptide release experiments, pre-TCs were assembled on MVHL-STOP mRNA in the presence of [^35^S]Met-tRNA_i_^Met^ and purified by SDG centrifugation as described above. 2 nM of purified pre-TC was rapidly mixed with the indicated combinations of 20 nM MoV-mon GTPase, 20 nM LV GTPase, 20 nM CTV1 GTPase, 20 nM HV-Sang GTPase, 20 nM MVMV GTPase, 20 nM human eRF3, 10 nM human eRF1, 10 nM human eRF1_Δmini-domain_, 10 nM Ac eRF1 and 10 nM MVMV eRF1 in buffer A supplemented with 0.2 mM GTP, 1 U/μl RiboLock RNase inhibitor (Thermo Scientific), and 0.5 mg/ml casein. Reactions were incubated at 37°C, and 20 μl aliquots were removed at the indicated times and immediately mixed with 980 μl of a mixture of 5% TCA and 0.75% casaminoacids. Samples were incubated on ice for 10 min and then centrifuged for 10 min at 20 000 g at 4°C. 800 μl aliquots from the top of each tube were collected, and the amount of released [^35^S]MVHL tetrapeptide was determined by scintillation counting. The resulting curves were fitted to a single exponential function using GraphPad Prism 7 software.

### Sequence alignment and Phylogenetic analysis

The sequences of translational GTPases and eRF1 moieties were aligned using CLUSTAL W. eRF1 open reading frames were identified in tBLASTn (https://blast.ncbi.nlm.nih.gov/Blast.cgi) searches of viral genomes, using known eRF1 sequences as seeds, and potential frame-shift and stop codon read-through sites were mapped by conceptual 3-frame translation of relevant segments of viral genomes.

For maximum likelihood (ML) phylogenetic analysis of translational GTPases encoded by giant viruses, *Homo sapiens* and *Acanthamoeba castellani* Neff, sequences were submitted to the web server phylogeny.lirmm.fr for alignment using CLUSTAL W (default parameters) and elimination of positions containing gaps (54). The phylogenetic tree was inferred in PhyML 3.0 (default parameters) (55) using these trimmed and aligned sequences, and applying the WAG model for amino acid substitution and the approximate likelihood-ratio test (aLRT) as a statistical test for branch support (56). The resulting tree was visualized in TreeDyn (57), and fonts were adjusted in a graphical editor.

## RESULTS

### Two groups of trGTPases encoded by giant viruses

**Figure 1.**
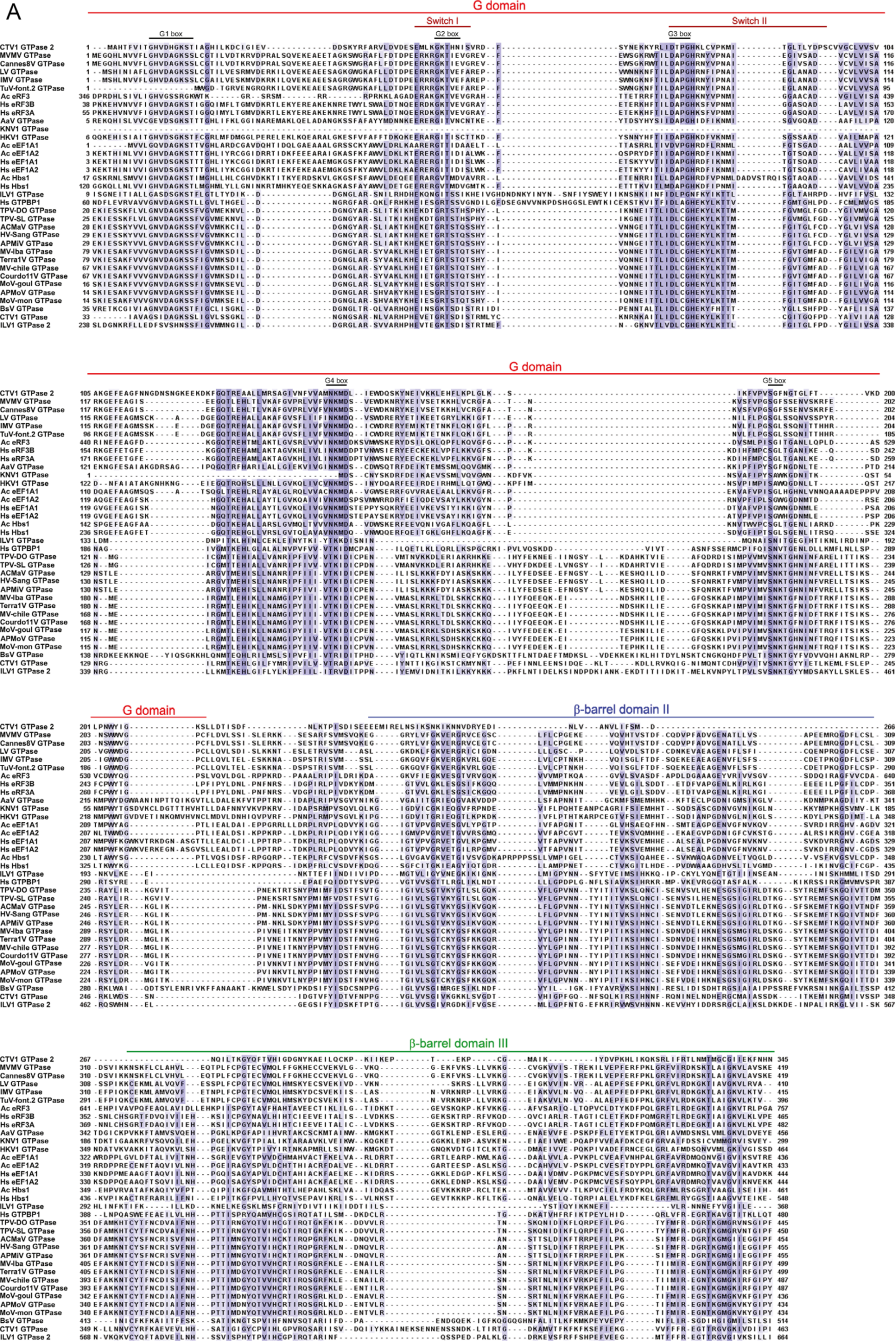

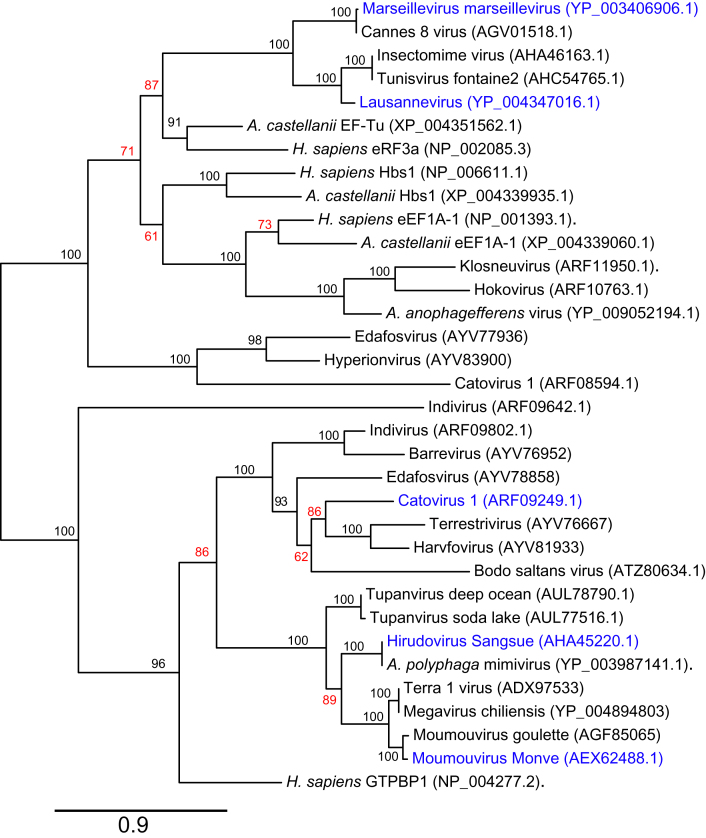
Multiple sequence alignment and phylogenetic tree of *H. sapiens* eEF1A1, eEF1A2, eRF3A, eRF3B, Hbs1 and GTPBP1, *Acanthamoeba castellanii* eEF1A1, eEF1A2, eRF3 and Hbs1, and predicted trGTPases encoded by giant viruses. (**A**) The multiple sequence alignment was made with ClustalW. NCBI accession numbers for *H. sapiens* proteins: eEF1A1 (NP_001393), eEF1A2 (NP_001949), GTPBP1 (NP_004277), eRF3A (NP_002085), eRF3B (NP_060564), HBS1 (NP_006611). NCBI accession numbers for *Acanthamoeba castellanii* proteins: eEF1A1 (XP_004339060), eEF1A2 (XP_004334950), eRF3 (XP_004351562) and HBS1 (XP_004339935). NCBI accession numbers for viral GTPases: *A. castellanii mamavirus* (ACMaV) (AEQ60824), *Hirudo virus strain Sangsue* (HV-Sang) (AHA45220), *A. polyphaga mimivirus* (APMiV) (YP_003987141), *Megavirus lba* (MV-lba) (AGD92699), *Terra1 virus* (Terra1V) (ADX97533), *Megavirus chilensis* (MV-chile) (YP_004894803), *Courdo11 virus* (Courdo11V) (ADX97532), *Moumouvirus goulette* (MoV-goul) (AGF85065), *A. polyphaga moumouvirus* (APMoV) (ADX97534), *Moumouvirus monve* (MoV-mon) (AEX62488), *Marseillevirus marseillevirus* (MVMV) (YP_003406906), *Cannes 8 virus* (Cannes8V) (AGV01518), *Lausannevirus* (LV) (YP_004347016), *Insectomime virus* (IMV) (AHA46163), *Tunisvirus fontaine2* (TuV-font.2) (AHC54765), *Aureococcus anophagefferens virus* (AaV) (YP_009052194), *Klosneuvirus* (KNV1) (ARF11950), *Indivirus* (ILV1) (GTPase: ARF09642; GTPase2: ARF09802), *Catovirus* (CTV1) (GTPase: ARF09249; GTPase2: ARF08594), *Tupanvirus deep ocean* (TPV-DO) (AUL78790), *Tupanvirus soda lake* (TPV-SL) (AUL77516), *Hokovirus* (HKV1) (ARF10763), *Bodo saltans virus* (BsV) (ATZ80634). Domains, Switch I and Switch II elements and conserved G motifs (24) are indicated. GTPases encoded by *A. castellanii mamavirus, Hirudo virus strain Sangsue, A. polyphaga mimivirus, Megavirus lba, Terra1 virus, Megavirus chilensis, Courdo11 virus, Moumouvirus goulette, A. polyphaga moumouvirus, Moumouvirus monve, Catovirus, Indivirus* (GTPase and GTPase2) Tupanvirus deep ocean, Tupanvirus soda lake and Bodo saltans virus formed a group that was closer to GTPBP1, whereas the second group of GTPases encoded by *Catovirus* (GTPase2), *Aureococcus anophagefferens virus, Klosneuvirus, Marseillevirus marseillevirus, Cannes 8 virus, Lausannevirus, Insectomime virus*, and *Tunisvirus fontaine2* were closer to eRF3, Hbs1 and eEF1A. (**B**) Maximum Likelihood phylogenetic analysis of translational GTPases encoded by giant viruses, *Homo sapiens* and *Acanthamoeba castellani* Neff (a free-living amoeba). The phylogenetic tree was derived using PhyML 3.0 (55) and aligned sequences as indicated. The approximate likelihood ratio test (aLRT) was used as a statistical test for branch support; an alRT score of ≥90 is consistent with a bootstrap score of at least 75 (56). Poorly supported nodes with scores of <90 are shown in red. Translational GTPases chosen for biochemical analysis are shown in blue.

Giant viruses encode multiple translation-related genes that usually include one or more trGTPases. Viral trGTPases have the typical trGTPase domains and GTP binding elements, and sequence alignment suggested that EF1 family members cluster into two distinct groups (Figure 1A). Maximum likelihood phylogenetic analysis done using PhyML (Figure 1B) (55) confirmed that viral GTPases segregate into two classes, one of which also contains eRF3, Hbs1 and eEF1A, and the other, GTPBP1. One group included proteins that were closer to eRF3, Hbs1 and eEF1A, whereas members of the second group are closer to GTPBP1. GTPBP1-like proteins are encoded exclusively by members of *Mimiviridae*, whereas eRF3/Hbs1/eEF1A-related proteins are encoded predominantly by members of *Marseilleviridae*, but also by some members of Mimiviridae, such as *Aureococcus anophagefferens* virus (9), as well as Klosneuviruses (e.g. Klosneuvirus and Catovirus) which encode a large repertoire of translation related genes (10). Indeed, Catovirus encodes members of both classes: CTV1 GTPase in the first, and CTV2 GTPase in the second.

**Figure 2.**
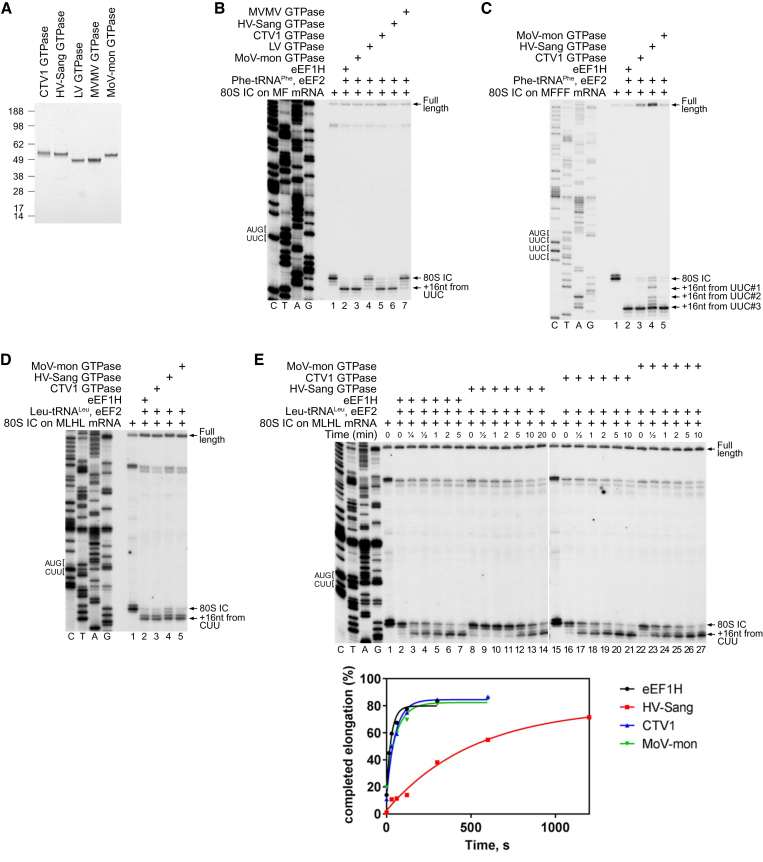
The eEF1A-like activity of Mimivirus HV-Sang, CTV1 and MoV-mon GTPases. (**A**) Purified His-tagged recombinant CTV1, HV-Sang, LV, MVMV and MoV-mon GTPases resolved by SDS-PAGE. (B–D) The activities of eEF1H and MVMV, HV-Sang, CTV1, LV and MoV- mon GTPases in (B and D) one-cycle and (C) three-cycle elongation on 80S initiation complexes (80S ICs) assembled on (**B**) MF-STOP, (**C**) MFFF-STOP and (**D**) MLHL-STOP mRNAs in the presence of eEF2 and (B, C) cognate native yeast Phe-tRNA^Phe^ and (D) cognate native Leu-tRNA^Leu^, assayed by toe-printing. (**E**) Time courses of one-cycle elongation by eEF1H and HV-Sang, CTV1 and MoV-mon GTPases on 80S ICs (assembled on MLHL-STOP mRNA) with cognate native Leu-tRNA^Leu^, assayed by toe-printing (upper panel), the efficiency of elongation was quantified by Phosphorimager (lower panel). (B–E) Positions of the ORF codons are shown on the left. Positions of the 80S ICs and elongation complexes are indicated by arrows on the right. Lanes C/T/A/G depict corresponding DNA sequences.

Since viral GTPBP-like proteins from distinct groups might differ functionally, we decided to characterize several proteins from each group. Five proteins were selected for further biochemical testing and were expressed and purified from *E. coli* (Figure 2A). Three proteins were taken from the GTPBP1-like group: Catovirus (CTV1) GTPase, Hirudo virus strain Sangsue (HV-Sang) GTPase, and Moumouvirus monve (MoV-mon) GTPase. These proteins had 49–51% similarity to human GTPBP1 but only 20–41% similarity to human eEF1A, eRF3 and Hbs1. The two remaining proteins were taken from the eEF1A/Hbs1/eRF3-like group: Lausannevirus (LV) GTPase, and Marseillevirus marseillevirus (MVMV) GTPase. These proteins had 50–56% similarity to human eEF1A, eRF3 and Hbs1 and 41–42% similarity to human GTPBP1.

### Mimivirus HV-Sang, CTV1 and MoV-mon GTPases possess eEF1A-like activity in translation elongation

Since GTPBP1 was found to possess eEF1A-like activity (34), all viral trGTPases were initially tested for a potential function in translation elongation using an *in vitro* reconstitution approach, in which ribosomal complexes are assembled from individual purified translational components. First, 80S initiation complexes were formed from 40S and 60S ribosomal subunits, initiation factors and Met-tRNA_i_^Met^ on derivatives of β-globin mRNA containing a β-globin 5′UTR and a short (2–4 codons) open reading frame (ORF) followed by a UAA stop codon. Elongation was then induced by addition of eEF2, specific cognate aa-tRNAs and eEF1H or a viral trGTPase, after which the ribosomal position on mRNA was determined by toe-printing.

In single-cycle elongation on the UUC (Phe) codon with cognate native yeast Phe-tRNA^Phe^, the CTV1, HV-Sang and MoV-mon GTPBP1-like GTPases had an activity that was comparable to that of eEF1H, whereas the LV and MVMV eEF1A/Hbs1/eRF3-like GTPases were inactive (Figure 2B). Thus, this translation elongation assay revealed a clear distinction between the two GTPase groups, pointing to a functional significance to the phylogenetic division identified by computational analysis. CTV1 and MoV-mon GTPases were also able to promote efficient elongation on three consecutive UUC (Phe) codons, whereas the activity of the HV-Sang GTPase in three-cycle elongation was lower (Figure 2C). The HV-Sang GTPase was also more sensitive to post-transcriptional modifications of tRNA. Thus, all three viral GTPBP1-like GTPases promoted efficient elongation on the CUU (Leu) codon with native Leu-tRNA^Leu^ tRNA (Figure 2D), but the activity of the HV-Sang GTPase was almost abolished when native Leu-tRNA^Leu^ was replaced by the *in vitro* transcript, whereas CTV1 and MoV-mon GTPases remained active (Supplementary Figure S1A). To further compare the elongation activities of viral GTPases and eEF1H, we performed time-course single-cycle elongation experiments on the CUU (Leu) codon with native Leu-tRNA^Leu^. In this assay, CTV1 and MoV-mon GTPases and eEF1H promoted elongation with similar rates, whereas elongation mediated by the HV-Sang GTPase was substantially slower (Figure 2E). Thus, in all experiments, the activity of the HV-Sang GTPase was lower than that of CTV1 and MoV-mon GTPases, suggesting a higher divergence of the HV-Sang GTPase from human eEF1H.

Consistent with the eEF1A-like activity of CTV1, MoV-mon and HV-Sang GTPases in elongation, their UV cross-linking to GTP was stimulated by aa-tRNA (Supplementary Figure S1B, upper panel). Similarly to eEF1A, but unlike GTPBP1 (34), interaction of CTV1, MoV-mon and HV-Sang GTPases with GTP was not stimulated by deacylated tRNA (Supplementary Figure S1B, upper panel). In contrast, cross-linking to GTP of the LV and MVMV eEF1A/Hbs1/eRF3-like GTPases was not affected by either aminoacylated or deacylated tRNAs (Supplementary Figure S1B, lower panel). Again, consistent with their elongation activity, GTP hydrolysis by CTV1, MoV-mon and HV-Sang GTPases was stimulated by establishment of codon-anticodon interaction (i.e. by the simultaneous presence of elongation complexes and cognate aa-tRNAs), despite rather high background levels of GTP hydrolysis that occurred in the presence of aa-tRNA alone (Supplementary Figure S1C).

### Marseillevirus LV and MVMV GTPases possess the eRF3-like activity in translation termination

**Figure 3.**
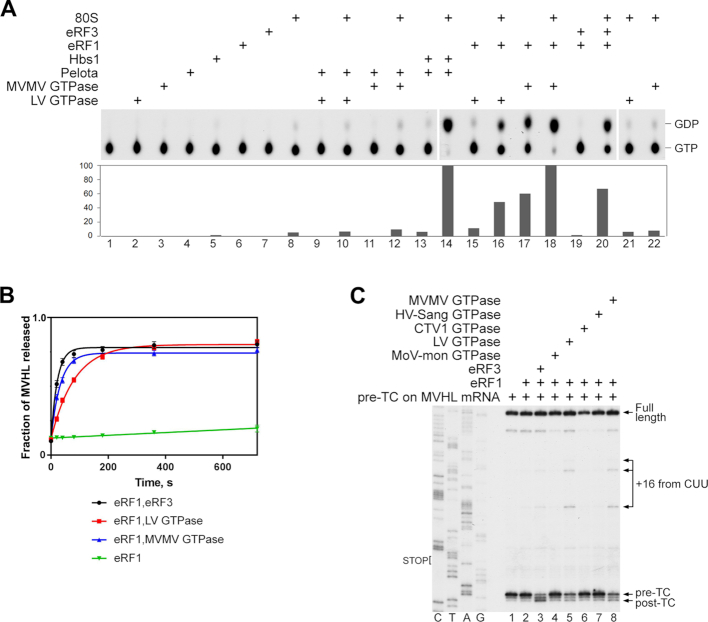
The eRF3-like activity of Marseillevirus LV and MVMV GTPases. (**A**) TLC analysis of [α-^32^P]GTP hydrolysis by LV and MVMV GTPases in the presence/absence of different combinations of mammalian 80S ribosomes, human eRF1 and human Pelota, as indicated. In control experiments, [α-^32^P]GTP is hydrolyzed by human eRF3 in the presence of 80S ribosomes and eRF1, and by human Hbs1 in the presence of 80S ribosomes and Pelota. The efficiency of hydrolysis was quantified by Phosphorimager and normalized to the condition with the highest GTP hydrolysis. (**B**) Kinetics of [^35^S]MVHL tetrapeptide release by eRF1 alone (green), eRF1 and eRF3 (black), eRF1 and LV GTPase (red), or eRF1 and MVMV GTPase (blue). The concentrations of pre-TCs, eRF1 and eRF3/LV/MVMV GTPases were 2, 10 and 20 nM, respectively. Each time point represents the average of three independent experiments. Error bars represent SD. (**C**) Toe-printing analysis of post-termination complexes (post-TCs) obtained by incubation of pre-termination complexes (pre-TCs) formed on MVHL-STOP mRNA with eRF1 alone or in combination with eRF3, MoV-mon GTPase, LV GTPase, CTV1 GTPase, HV-Sang GTPase or MVMV GTPase, as indicated. The positions of pre-TCs, post-TCs, full-length cDNA and migrated upstream post-termination ribosomes (+16 from CUU) are shown on the right. Lanes C/T/A/G depict the corresponding DNA sequence.

Since LV and MVMV GTPases are phylogenetically related to eEF1A/eRF3/Hbs1 but did not have eEF1A-like activity, they were tested for the eRF3-like and Hbs1-like activities. GTP hydrolysis by eRF3 and Hbs1 is induced by the simultaneous presence of 80S ribosomes and their binding partners, eRF1 and Pelota, respectively (27,29,31,32,58). We therefore assayed the ability of mammalian 80S ribosomes, eRF1 and Pelota to stimulate GTP hydrolysis by LV and MVMV GTPases. As with eRF3, the GTPase activity of the LV and MVMV GTPases was induced by the simultaneous presence of vacant 80S ribosomes and human eRF1, but not Pelota (Figure 3A, compare lanes 9–12 with lanes 15–18). Interestingly, unlike human eRF3, GTP hydrolysis by viral GTPases could also be stimulated by eRF1 alone, particularly strongly in the case of the MVMV GTPase (Figure 3A, lanes 15, 17 and 19). The ribosome-uncoupled activity of the MVMV GTPase was specific to GTP and was not due to a contaminating NTPase (Supplementary Figure S2A). As expected, the mimivirus eEF1A-like proteins (HV-Sang, CTV1 and MoV-mon GTPases) had no additional Hbs1-like or eRF3-like activity in the GTP hydrolysis assay (Supplementary Figure S2B), further confirming the functional segregation of the two viral protein groups.

Next, we investigated the activities of LV and MVMV GTPases in stimulation of peptide release by eRF1. For this, we used *in vitro* reconstituted mammalian pre-termination complexes (pre-TCs) formed on MVHL-STOP mRNA (31) and containing tRNA^Leu^ linked to a [^35^S]MVHL tetrapeptide in the P site and a UAA stop codon in the A site. Termination was induced using a low concentration of eRF1, which allows efficient peptide release only in the presence of eRF3 (31). Consistently, eRF1 alone had no significant peptide release activity (Figure 3B, green). In these conditions, LV and MVMV GTPases were able to stimulate peptide release by human eRF1 with efficiencies that were similar to that of human eRF3 (Figure 3B, black, red and blue). In contrast, the mimivirus eEF1A-like proteins (HV-Sang, CTV1 and MoV-mon GTPases) had no influence on the activity of eRF1 in this assay (Supplementary Figure S2C).

The termination activities of LV and MVMV GTPases were further assayed by toe-printing. The duration of incubation of pre-TCs formed on MVHL-STOP mRNA with eRF1 and eRF3-like GTPases before addition of the reverse transcriptase was long enough to complete peptide release. Binding of eRF1 to the A site of pre-TCs causes compaction of mRNA (59) that results in the appearance of the characteristic +2nt toe-print shift, and since eRF1 remains associated with ribosomal complexes after peptide release, this shift persists in post-termination complexes (post-TCs) (31). As expected, a strong +2nt toe-print shift was observed after incubation of pre-TCs with human eRF1/eRF3 (Figure 3C, lane 3). On the other hand, incubation of pre-TCs with eRF1 and LV or MVMV GTPases yielded much weaker +2nt toe-print shifts, but led to the appearance of more prominent additional stops +16 nt from the upstream CUU codons that were cognate to the P site tRNA^Leu^ (Figure 3C, lanes 5 and 8). Migration of post-termination ribosomes to nearby codons that are cognate to the P site tRNA has been reported previously and was attributed to destabilization of the P site codon–anticodon interaction due to adoption by the P site deacylated tRNA of the P/E hybrid hybrid state, particularly after losing the stabilizing effect of eRF1 due to its dissociation (60). Thus, although viral eRF3-like GTPases strongly stimulate peptide release by human eRF1, the binding between eRF1/viral eRF3-like GTPase complexes and the post-termination ribosomes is not stable, resulting in substantial ribosome migration. As expected, no changes in toe-print pattern was observed after incubation of pre-TCs with eRF1 and eEF1A-like viral GTPases (Figure 3C, lanes 4, 6 and 7).

### The influence of eRF1’s nature on the activities of Marseillevirus LV and MVMV eRF3-like GTPases and human eRF3

**Figure 4.**
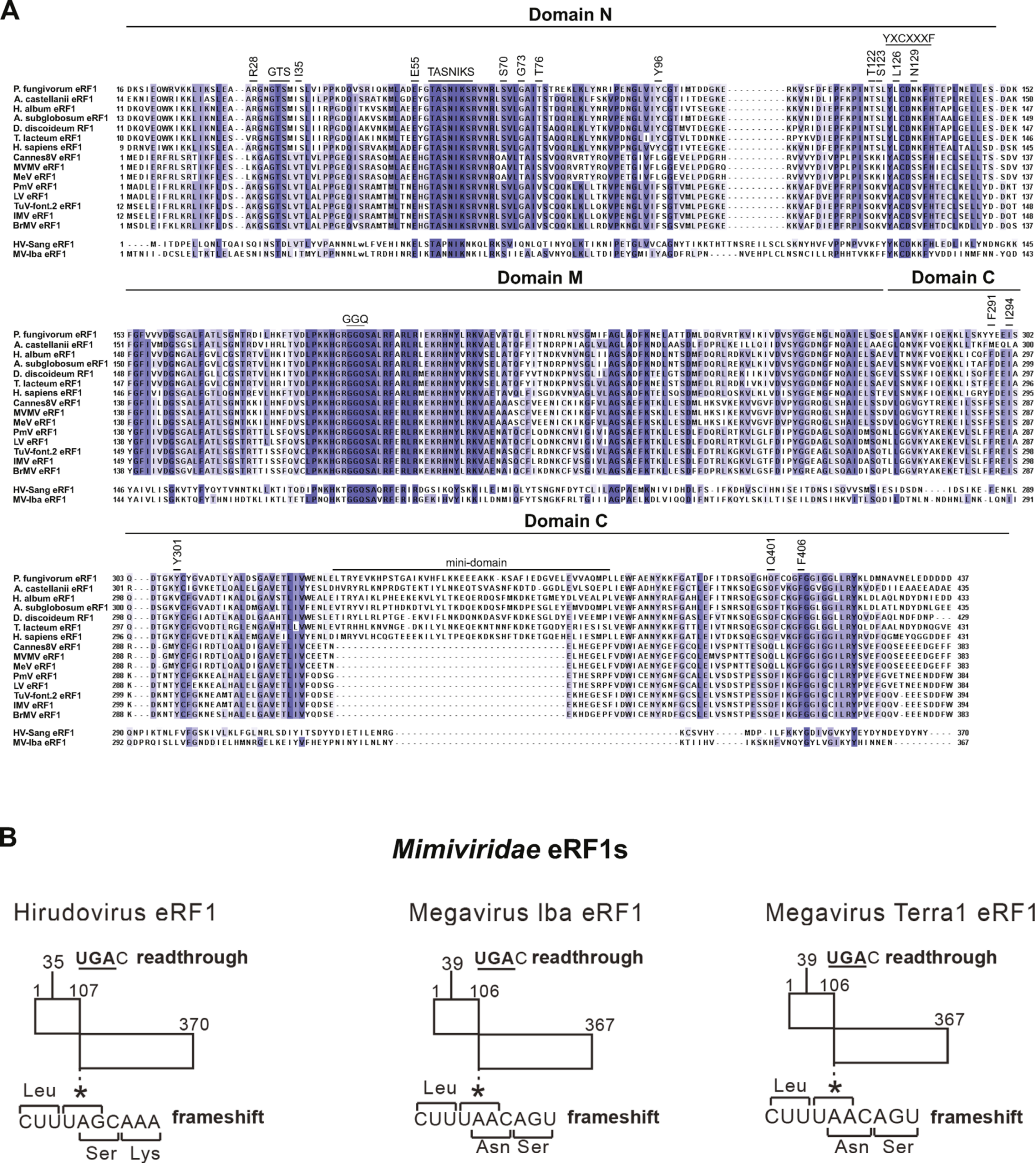
Multiple sequence alignment of eRF1 encoded by different eukaryotes and by giant viruses. (**A**) The alignment was made with Clustal W. NCBI accession numbers for the eukaryotic eRF1 proteins: *H. sapiens* (NP_004721), *A. castellanii* (XP_004338193), *D. discoideum* (XP_636638), *P. fungivorum* (PRP77657), *H. album* (XP_020437748), *A. subglobosum* (XP_012752976), *T. lacteum* (KYR01314). NCBI accession numbers for eRF1 encoded by giant viruses: *Marseillevirus marseillevirus* (MVMV) (ADB03912), *Melbournevirus* (MeV) (AIT54729), *Cannes 8 virus* (Cannes8V) (AGV01484), *Tunisvirus fontaine2* (TuV-font.2) (AHC54787), *Insectomime virus* (IMV) (AHA46142), *Port-miou virus* (PMV) (ALH06844), *Lausannevirus* (LV) (AEA07018), *Brazilian marseillevirus* (BrMV) (AMQ10707), *Hirudo virus strain Sangsue* (HV-Sang) (AHA45107.1, AHA45106.1 and intervening/upstream sequences) and *Megavirus lba* (MV-lba) (AGD92187.1, AGD92188.1 and the intervening sequence). Domains (N, M, C and mini-domain), and the positions of conserved sequence motifs and amino acid residues involved in stop codon recognition and peptide release (GTS_31–33_, E_55_, TASNIKS_58–64_, YxCxxxF_125–131_, GGQ_183–185_) (*H. sapiens* residue numbering), amino acid residues responsible for eRF3 binding (F_291_, I_294_, Y_301_, Q_397_, Q_401_, F_406_) (*H. sapiens* residue numbering) (61), and amino acid residues that could influence eRF1’s stop codon decoding specificity (R_28_, I_35_, S_70_, G_73_, T_76_, Y_96_, T_122_, S_123_, L_126_, N_129_) (*H. sapiens* residue numbering) (62) are indicated. (**B**) Schematic representations of examples of Mimivirus eRF1 coding regions, showing the potential for synthesis of full-length proteins by the combination of readthrough of termination codons and frame-shifting.

Giant viruses with genomes that encode eEF1A/eRF3/Hbs1-like GTPases also encode eRF1 homologues. Thus, in the cell, viral eRF3-like GTPases would likely encounter amoebal and viral eRF1s. To investigate the activities of eRF3-like LV and MVMV GTPases with their biologically relevant partners, we first compared amino acid sequences of eRF1s from a variety of giant viruses and eukaryotic organisms (Figure 4A). eRF1 is a highly conserved eukaryotic factor, and the human and amoebal homologs have 85% similarity. Marseillevirus eRF1s also show a high degree of conservation with their eukaryotic counterparts and contain all conserved sequence motifs involved in stop codon recognition and peptide release (GTS_31–33_, E_55_, TASNIKS_58–64_, YxCxxxF_125–131_, GGQ_183–185_; *H. sapiens* residue numbering) and the majority of the amino acids responsible for binding to eRF3 (F_291_, I_294_, Y_301_, Q_401_, F_406_; *H. sapiens* residue numbering) (61). However, they have amino acid residues at many positions that match substitutions that influence the stop codon decoding capacity of human eRF1 (R_28_, I_35_, S_70_, G_73_, T_76_, Y_96_, T_122_, S_123_, L_126_, N_129_; *H. sapiens* residue numbering) (62). Moreover, viral eRF1s lack the mini-domain, a flexible insertion into the rigid core of the C-terminal domain that is involved in interaction with eRF3 (63,64). The mini-domain stabilizes the interaction of eRF1 with the 40S subunit (65,66) and mutations in it affect stop codon specificity and enhance termination on UAG (67). Interestingly, genomes of some species of *Mimiviridae* also encode eRF1s, but their reading frames are interrupted and their expression would require read-through and frameshifting to bypass premature stop codons (Supplementary Table S1; Figure 4B; Supplementary Figure S3) (14). Mimivirus eRF1s diverge more from the eukaryotic consensus sequence, as they lack some or all of the mini-domain, are C-terminally truncated, so that they commonly lack equivalents of F_406_ and some other determinants of the eRF1-eRF3 interaction (e.g. acidic residues in the C-terminal tail) (63,64,68), and commonly have substitutions in GTS and TASNIKS motifs (shown for Hirudo virus strain Sangsue and Megavirus lba eRF1 in Figure 4A).

**Figure 5.**
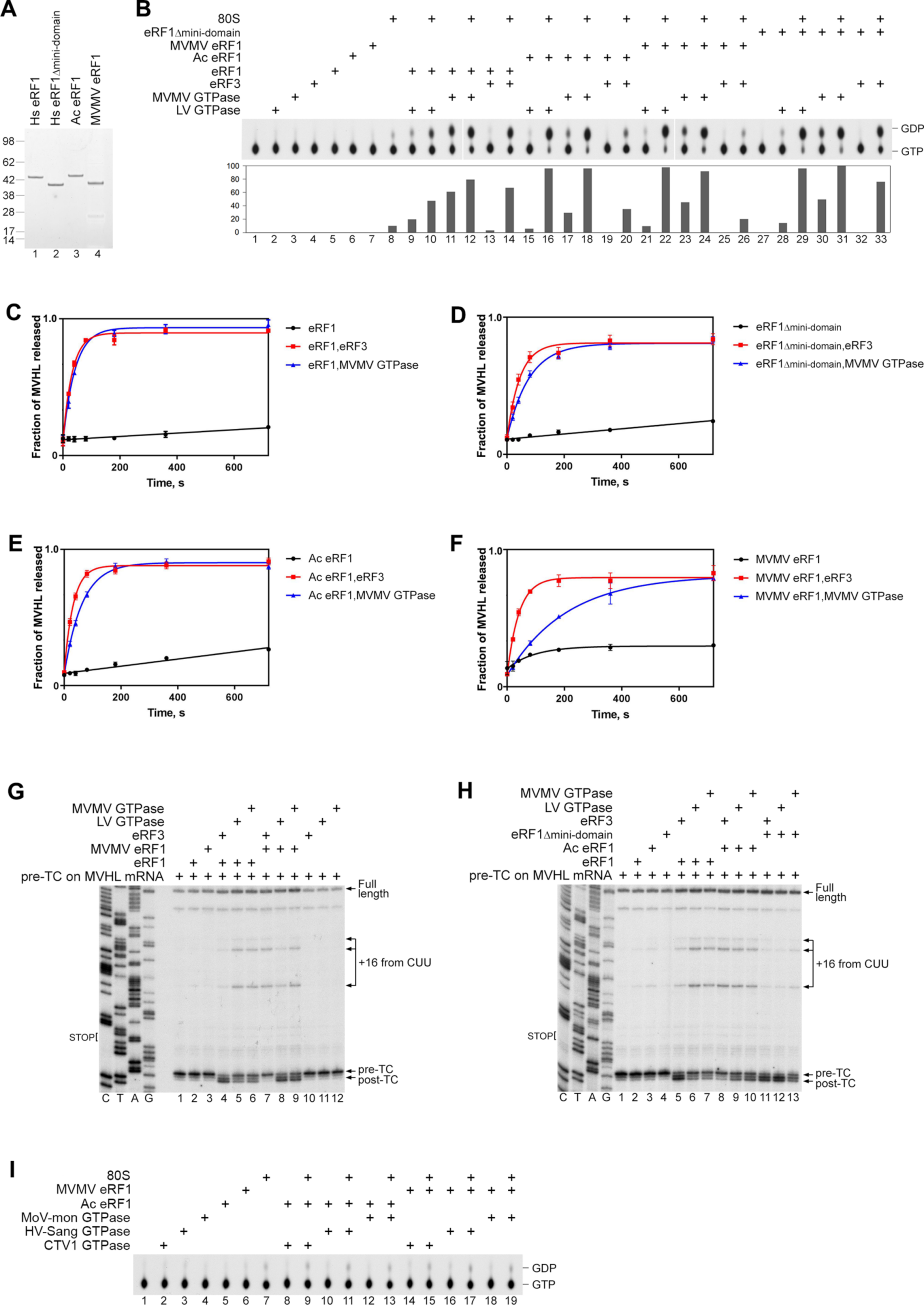
The activities of Marseillevirus LV and MVMV GTPases and human eRF3 with various eRF1 partners. (**A**) Purified recombinant human *wt* eRF1, human eRF1_Δmini-domain_, *A. castellanii* eRF1 and MVMV eRF1, resolved by SDS-PAGE. (**B**) TLC analysis of [α-^32^P]GTP hydrolysis by LV GTPase, MVMV GTPase and eRF3 in the presence/absence of different combinations of 80S ribosomes, human *wt* eRF1, human eRF1_Δmini-domain_, *A. castellanii* eRF1 and MVMV eRF1, as indicated. The efficiency of hydrolysis was quantified by Phosphorimager and normalized to the condition with the highest GTP hydrolysis. (C–F) Kinetics of [^35^S]MVHL tetrapeptide release by (**C**) human *wt* eRF1, (**D**) human eRF1_Δmini-domain_, (**E**) *A. castellanii* eRF1 and (**F**) MVMV eRF1 alone (black) or in the presence of human eRF3 (red) or MVMV GTPase (blue). The concentrations of pre-TCs, eRF1s and eRF3/MVMV GTPases were 2 nM, 10 nM and 20 nM, respectively. Each time point represents the average of three independent experiments. Error bars represent SD. (**G, H**) Toe-printing analysis of post-termination complexes (post-TCs) obtained by incubation of pre-termination complexes (pre-TCs) formed on MVHL-STOP mRNA with human *wt* eRF1, human eRF1_Δmini-domain_, *A. castellanii* eRF1 or MVMV eRF1 alone or in the presence of human eRF3, LV GTPase, or MVMV GTPase, as indicated. The positions of pre-TCs, post-TCs, full-length cDNA and migrated upstream post-termination ribosomes (+16 from CUU) are shown on the right. Lanes C/T/A/G depict corresponding DNA sequences. (**I**) TLC analysis of [α-^32^P]GTP hydrolysis by MoV-mon, HV-Sang and CTV1 GTPase in the presence/absence of combinations of 80S ribosomes, *A. castellanii* eRF1 and MVMV eRF1, as indicated.

To determine how the nature of eRF1 affects the activities of viral LV and MVMV GTPases and human eRF3, amoebal *A. castellanii* eRF1, MVMV eRF1 and human *wt* eRF1 and eRF1 mutant lacking the mini-domain (eRF1_Δmini-domain_) were expressed and purified from *E. coli* (Figure 5A). First, we compared the ability of these eRF1s to stimulate the GTPase activity of LV and MVMV GTPases and human eRF3 in the presence and in the absence of mammalian 80S ribosomes (Figure 5B). Human eRF3 showed substantially higher GTPase activity in the presence of 80S ribosomes and human *wt* and mutant eRF1 than 80S ribosomes and *A. castellanii* or MVMV eRF1 (Figure 5B, compare lanes 13–14 and 32–33 with lanes 19–20 and 25–26). No 80S-uncoupled GTP hydrolysis by human eRF3 was observed in the presence of any eRF1. In the case of LV and MVMV GTPases, the lowest 80S-dependent and the highest 80S-uncoupled GTPase activities were observed in the presence of human *wt* eRF1 (Figure 5B, lanes 9–12). Replacement of human eRF1 by *A. castellanii* or viral MVMV eRF1s stimulated 80S-dependent GTP hydrolysis, and importantly, substantially reduced the 80S-uncoupled GTPase activity of viral GTPases, particularly in the case of the MVMV GTPase (Figure 5B, lanes 15–18 and 21–24). Thus, combining human eRF3 and viral eRF3-like GTPases with their biologically relevant partners increased the efficiency and specificity of ribosome-dependent GTP hydrolysis. Interestingly, deletion of the mini-domain in human eRF1 reduced the 80S-uncoupled and stimulated the 80S-dependent GTPase activity of LV and MVMV GTPases (Figure 5B, lanes 28–31). The fact that *A. castellanii* eRF1, which contains the mini-domain, did not induce ribosome-independent GTP hydrolysis by viral GTPases, suggests the presence of additional features in human *wt* eRF1 that contribute to stimulation of 80S-uncoupled hydrolysis by viral GTPases.

Next, we compared the efficiency of peptide release by distinct eRF1s when they were combined with either human eRF3 or MVMV GTPase, using mammalian pre-TCs assembled on the MVHL-STOP mRNA. Human *wt* eRF1, eRF1_Δmini-domain_ and *A. castellanii* eRF1 released peptides at similar rates irrespective of whether they were combined with eRF3 or MVMV GTPase (Figure 5C–E). Surprisingly, viral MVMV eRF1 was less active with cognate MVMV GTPase than with human eRF3 (Figure 5F) even though MVMV eRF1 stimulated termination-uncoupled GTP hydrolysis by MVMV GTPase more strongly and specifically than human eRF3 (Figure 5B, compare lanes 23–24 and 25–26). Peptide release by individual eRF1s was very inefficient in all cases (Figure 5C–F).

To assess the stability of interaction of different eRF1/eRF3-like pairs with mammalian post-TCs, we employed the toe-printing assay. The duration of incubation of pre-TCs formed on MVHL-STOP mRNA with eRF1/eRF3-like pairs before addition of reverse transcriptase was sufficient for complete peptide release in all cases. Stable interaction with post-TCs was observed for the human eRF1/eRF3 pair, which was manifested by the appearance of the strong +2nt toe-print shift and only minor ribosome migration (Figure 5G, lane 4; Figure 5H, lane 5). When human eRF1 was combined with LV or MVMV GTPases, ribosomal association of factors with post-TCs was less stable, resulting in reduction of the +2nt toe-print shift and enhancement of ribosome migration (Figure 5G, lanes 5–6; Figure 5H, lanes 6–7). In contrast, interaction of MVMV eRF1 with post-TCs was more stable with LV and MVMV GTPases than with human eRF3 (Figure 5G, compare lane 7 with lanes 8–9). In the case of *A. castellanii* eRF1, slightly more stable interaction with post-TCs was also observed when it was incubated with LV and MVMV GTPases than with human eRF3 (Figure 5H, compare lane 8 with lanes 9–10). Thus, in all cases, more stable interaction with post-TCs occurred when eRF1s were combined with their biologically relevant eRF3-like partners. Interestingly, in the case of the eRF1_Δmini-domain_ mutant, interaction with post-termination ribosomes was stable in the case of both human eRF3 and viral LV and MVMV GTPases (Figure 5H, lanes 11–13). This effect is consistent with the positive influence of deletion of the mini-domain on the GTPase activity of the MVMV GTPase (Figure 5B). Taken together, our data on 80S-independent GTP hydrolysis and stability of post-TCs reveal some degree of functional incompatibility between heterologous eRF1s and eRF3-like GTPases, and conversely, of complementarity between viral factors. They also show adaptation of the viruses to their amoebal hosts.

Although mimiviral CTV1, HV-Sang and MoV-mon GTPases did not function in termination with human eRF1 (Figure 3C and S2C), considering the omnipotent role of archaeal elongation factor 1 alpha in elongation, termination and quality control (e.g. 69), we tested whether mimiviral trGTPases could display eRF3-like activity with biologically more relevant partners, i.e. *A. castellanii* or MVMV eRF1s. However, in contrast to MVMV or LV GTPases, the GTPase activity of CTV1, HV-Sang and MoV-mon GTPases was not stimulated by the simultaneous presence of mammalian 80S ribosomes and either *A. castellanii* or MVMV eRF1s (Figure 5I), arguing against an omnipotent function of these GTPases in translation elongation and termination.

## DISCUSSION

Here, we report that giant virus trGTPases belonging to the EF1 family segregate into two classes, one more closely related to eEF1A/Hbs1/eRF3 and the other to GTPBP1. *Marseilleviridae* encode only proteins belonging to the first class, whereas most mimivirus proteins of this type belong to the second. A few members of the *Klosneuvirinae* subfamily of *Mimiviridae* diverge from this pattern: Bodo saltans virus encodes only a GTPBP1-like factor, but other members instead encode an eEF1A/Hbs1/eRF3-like factor (e.g. Klosneuvirus and Hokovirus), or even encode both types of trGTPase (e.g. Catovirus and Indivirus) (6,10). The presence of both classes of trGTPases in some but not all members of this subfamily reflects the complex patterns of gene acquisition and loss that characterize the evolution of giant viruses (3). All the viral trGTPases that were tested here are functional translation factors, but the two groups have very different activities. The GTPBP1-like *Mimiviridae* GTPases (exemplified by MoV-mon, HV-Sang and CTV1) possess an eEF1A-like elongation activity and deliver aa-tRNA into the ribosomal A site, while eEF1A/Hbs1/eRF3-like *Marseilleviridae* GTPases (exemplified by LV and MVMV) have an eRF3-like termination activity and stimulate peptide release by eRF1.

In addition to the trGTPases, the genomes of giant viruses contain different sets of other translation-related genes (16,17). Members of all the lineages of *Mimiviridae* that encode GTPBP1-type GTPases also encode variable combinations of tRNAs, tRNA modifying enzymes and aminoacyl-tRNA synthetases (Supplementary Table S1; 6,10,11) whose transcription is upregulated in conditions of limited nutrient availability (20). The fact that the GTPases from three distinct *Mimiviridae* lineages have an eEF1A-like elongation activity and that all mimiviruses encode factors related to the process of translation elongation suggest that other mimivirus GTPBP1-related GTPases likely also possess elongation activity.

**Figure 6.**
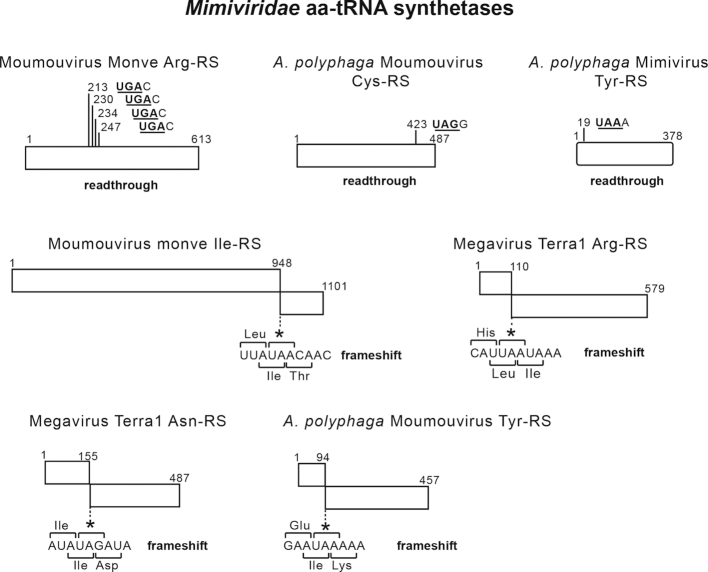
Mimivirus aminoacyl-tRNA synthetases encoded by readthrough and frame-shifting. Schematic representations of examples of Mimivirus aminoacyl-tRNA synthetase (aaRS) coding regions, showing the potential for synthesis of full-length proteins by readthrough of termination codons and frame-shifting.

The basis for the advantages that expression of virus-encoded components of the translation elongation apparatus could give to replication of mimiviruses has not been determined, but various possibilities that are not mutually exclusive can be considered. They include the ability to circumvent the putative shut-off of the host's translation system during infection by restoring levels of active components (17), and enhancement of viral replication by synthesis of translation factors that might be preferentially recruited to the ‘virus factories’ where viral protein synthesis, genome replication and capsid formation occur during infection (70). A further possibility is that encoding a complement of tRNAs and aminoacyl-tRNA synthetases might serve to expand the host range of giant viruses, enabling them to infect organisms in which the tRNA pool or the availability of factors are suboptimal for translation of viral mRNAs (e.g. 71). Indeed, the codon and amino acid usages of mimiviruses differ greatly from those of the *Acanthamoebae* species that are commonly used for their propagation (15), so that the ability to overcome deficiencies in the host tRNA pool could favor viral replication. Although mimivirus-encoded tRNAs do not generally correspond to codons that are used more frequently by mimiviruses than by *Acanthamoebae*, these species are likely not natural hosts for mimiviruses in which viral infection might benefit from expression of specific tRNAs or aminoacyl-tRNA synthetases (15). The possibility that this mechanism might favor viral replication in other host species can therefore not be excluded. A fourth possibility is that virus-encoded translation factors have become specialized following their capture from an ancestral host such that they now promote selective translation of specific viral mRNAs. For example, mimivirus eRF1 proteins and some mimivirus aminoacyl-tRNA synthetases are encoded by interrupted reading frames, and their expression would require read-through and frameshifting to bypass premature stop codons (Supplementary Table S1; Figures 4B and 6) (14). Analysis of recently characterized mimivirus genomes such as those of Bodo saltans virus (6), Edafosvirus and Terrestrivirus (7), Klosneuviruses (e.g. Catovirus, Hokovirus and Kloseneuvirus) (10) and Tupanviruses (11) shows that would also express eRF1 proteins if they could exploit these mechanisms (Supplementary Figure S3). Interestingly, the potential stop-codon read-through element in all these viruses is strongly conserved (**UGA**C, where the stop codon is bold and underlined) and corresponds to the sequence that supports the highest level of read-through in eukaryotes (72), whereas frame-shifting would occur at a consensus sequence (CUU**UAG**) that resembles the CUU**UGA** shift-site/stop codon cassette that promotes +1 frame-shifting in the bacterial polypeptide chain release factor 2 (73). An intriguing possibility is that by analogy with various mutant forms of eEF1A/EF-Tu that promote frameshifting (74), mimivirus GTPases might also promote stop-codon suppression and/or frameshifting to allow translation of those genes. Suppression of stop codons might be facilitated further by viral tRNA^Trp^, which is one of the most commonly encoded tRNAs in mimivirus genomes (15,16).

All lineages of *Marseilleviridae* encode trGTPases that are closely related to eEF1A/Hbs1/eRF3, and in addition, they also encode eRF1, but in contrast to mimiviruses, do not contain genes for tRNAs or aminoacyl-tRNA synthetases (17) (Supplementary Table S1). The facts that two trGTPases from *Marseilleviridae* (LV and MVMV) had an eRF3-like activity, that eRF1 from this virus family (MVMV) was also functional in termination, and that all members of *Marseilleviridae* encode eRF1 suggest that other viral eEF1A/Hbs1/eRF3-related GTPases likely also possess eRF3-like termination activity, implying that *Marseilleviridae* encode an eRF1-eRF3 functional pair.

Viral termination factors possess some distinct structural characteristics compared to eukaryotic eRF1/eRF3. Thus, viral eRF3-like GTPases do not contain the prion-like N-terminal domain of eukaryotic eRF3 and have relatively low conservation in the two β-barrel domains, but contain all the functionally important elements in the G domain. The most notable structural difference between Marseillevirus/Mimivirus and eukaryotic eRF1s is the absence of the flexible mini-domain (67) in domain C of the viral homologs. Mimivirus eRF1s are also C-terminally truncated, and consequently lack some elements that have been implicated in the functional interaction of eRF1 with eRF3 (63,64,68). Viral eRF1 proteins also differ from their cellular counterparts at numerous individual amino acid residues: thus, MVMV eRF1 (Figure 4) contains residues equivalent to substitutions that affect stop codon specificity in eukaryotic eRF1 (61,62). However, not all viral eRF1s have these substitutions, and there is a clear distinction between Marseillevirus eRF1s, in which the residues at these positions generally correspond to conservative substitutions vis-à-vis human eRF1, and Mimivirus eRF1s, in which these residues are either identical to those in human eRF1 or correspond to much more divergent substitutions than in Marseillevirus eRF1s. It is therefore possible that these two subgroups of viral eRF1s have somewhat different specificities. Indeed, phylogenetic analysis indicated that eRF1 from Marseilleviruses is eukaryotic-like, whereas eRF1 from Mimiviruses and members of *Asfarviridae* and the proposed *Pithiviridae* may have an archaeal origin (3), supporting the probable segregation of viral eRF1 proteins into different functional classes.

Interestingly, peptide release by the MVMV eRF1/eRF3 complex was substantially slower than by any other pair that was tested. This difference was unlikely a consequence of just the absence of the mini-domain in the MVMV eRF1, because deletion of mini-domain from human eRF1 did not affect the rate of peptide release, and because in combination with human eRF3, peptide release by MVMV eRF1 was also efficient. One possible explanation is that the viral MVMV eRF1/GTPase pair is the most heterologous for the mammalian pre-TCs, and the activity of viral factors could be higher on amoebal ribosomal complexes. However, as noted above, some viral eRF1 proteins, including MVMV, contain residues with the potential to affect stop codon specificity and thus to delay codon recognition. MVMV eRF1 contains additional differences at residues adjacent to motifs that are known to determine stop codon recognition and peptide release in eukaryotic eRF1 that could also contribute to delayed peptide release. Further analysis will be required to determine the mechanism that is responsible for the relatively slow peptide release kinetics of termination mediated by the MVMV eRF1/eRF3 complex, and whether the potential to form eRF1/eRF3 complexes with different combinations of viral and cellular factors that promote translation termination with different kinetics is exploited, for example, by regulated expression of viral factors, to dynamically regulate termination during infection.

In any case, a consequence of an altered preference towards certain stop codons could be exploited to favor stop codon read-through, allowing translation of contiguous ORFs that are separated by a stop codon or synthesis of protein variants with longer C-termini. eRF1 variants that delay termination at specific stop codons, for example, in some ciliated protozoa, are associated with ‘shifty stop frame-shifting’ (75), and the divergent eRF1 moieties encoded by members of *Marseilleviridae* could potentially promote a similar process, which appears to be required for translation of some Marseillevirus polypeptides. For example, translation of a putative restriction endonuclease that is encoded by a single open reading frame (ORF) in Insectomime virus (Genbank accession: AHA46057) is encoded by two ORFs in Lausannevirus (YP_004347214 and YP_004347215) and its translation would require frame-shifting after ORF1 (76). Similarly, a putative origin of replication-binding protein that is encoded by a single ORF in Tunisvirus fontaine 2 (YP_009506852.1) is encoded by two overlapping ORFs in Noumeavirus (YP_009345432.1 and YP_009345433.1) (77). Meaningful experimental analysis of potential differences in the stop codon specificity of viral eRF1s will require the development of appropriate *in vitro* and *in vivo* assay systems using amoebae to ensure an environment containing homologous ribosomal pre-termination complexes.

In conclusion, we have determined that several giant viruses encode translational EF1-family trGTPases that are active either in translation elongation or termination. The possibilities that these factors act in concert with other virus-encoded components of the translation apparatus (e.g. tRNAs, aa-tRNA synthetases and eRF1) and that they function in a divergent manner in specific circumstances or on specific viral mRNAs to promote frame-shifting or stop codon read-through merit further investigation.

## Supplementary Material

gkz296_Supplemental_File
