# Supplementary material for: Two classes of EF1-family translational GTPases encoded by giant viruses
Source: Nucleic Acids Res. 2019 Apr 24;47(11):5761–76. doi: 10.1093/nar/gkz296 (PMC6582330; doi:10.1093/nar/gkz296)
Supplement: gkz296_Supplemental_File [file gkz296_supplemental_file.pdf]

**Supplemental Information**

# Two classes of EF1-family translational GTPases encoded by giant viruses

Alexandra Zinoviev*, Kazushige Kuroha, Tatyana V. Pestova and Christopher U. T. Hellen*

Department of Cell Biology, SUNY Downstate Medical Center, Brooklyn, NY, USA

* Corresponding authors

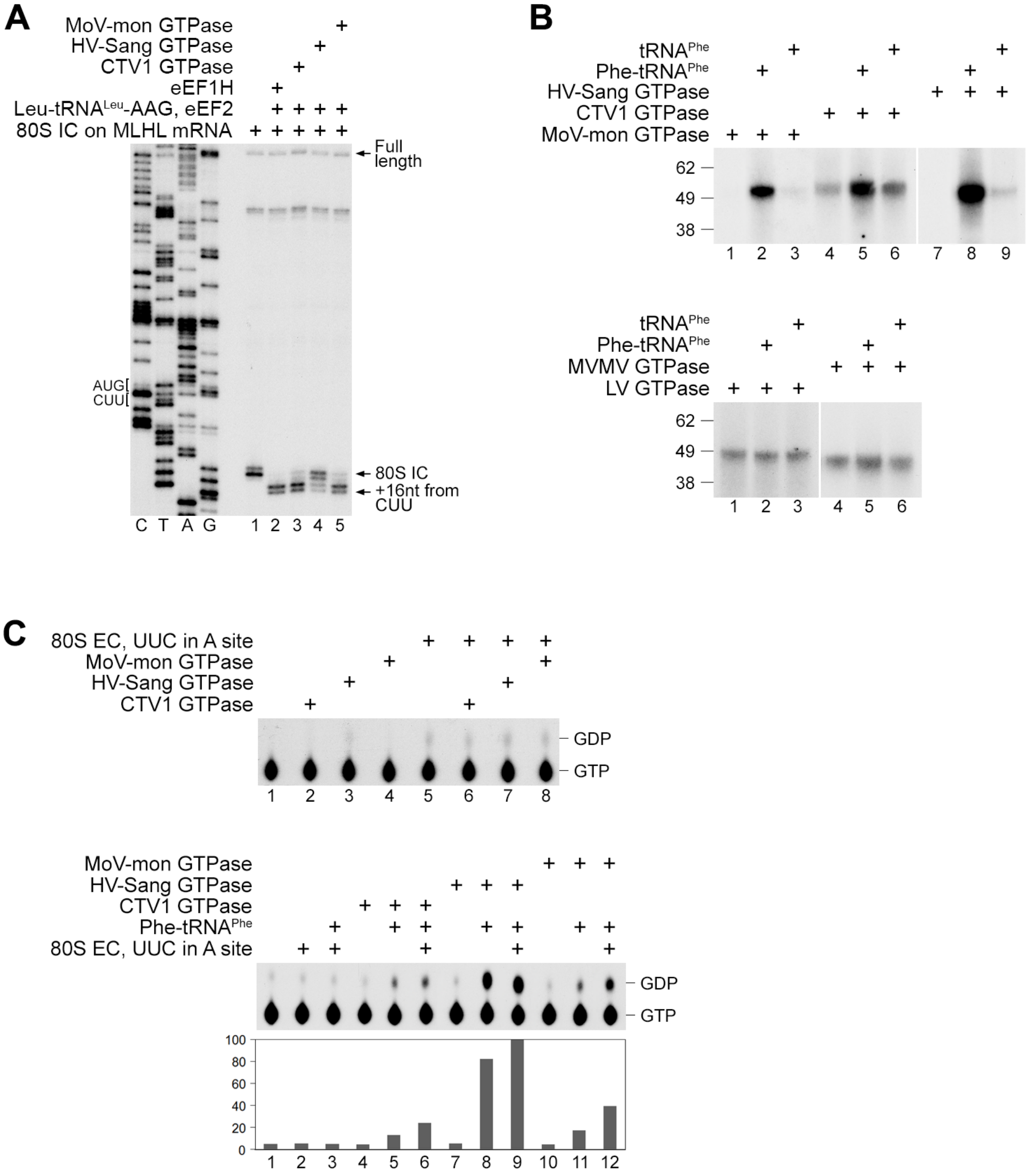


**Figure S1. The activities of CTV1, HV-Sang, MoV-mon, LV and MVMV GTPases in elongation, related to Figure 2.**

(A) The activities of eEF1H and CTV1, HV-Sang and MoV-mon GTPases in one-cycle elongation on 80S initiation complexes (80S ICs) assembled on MLHL-STOP mRNAs in the presence of eEF2 and cognate *in vitro* transcribed Leu-$tRNA^{Leu}$-AAG, assayed by toe-printing. Positions of the ORF codons are shown on the left. Positions of the 80SICs and elongation complexes are indicated by arrows on the right. Lanes C/T/A/G depict corresponding DNA sequences. (B) UV cross-linking of MoV-mon, CTV1 and HV-Sang GTPases (upper panel) and LV and MVMV GTPases (lower panel) to [$\alpha$-$^{32}$P]GTP depending on the presence of amino acylated Phe-$tRNA^{Phe}$ and deacylated $tRNA^{Phe}$, as indicated. Cross-linked proteins were analyzed by SDS-PAGE followed by autoradiography. (C) GTP hydrolysis by CTV1, HV-Sang and MoV-mon GTPases depending on the presence of 80S elongation complexes (formed on MSSLLF-STOP mRNA and containing MSSLL-$tRNA^{Leu}$ in the P sate and the UUC codon in the A site) (upper panel), and 80S elongation complexes in combination with cognate Phe-$tRNA^{Phe}$ (lower panel), assayed by TLC followed by autoradiography. The efficiency of GTP hydrolysis was quantified by Phosphorimager and normalized to the condition with the highest GTP hydrolysis.

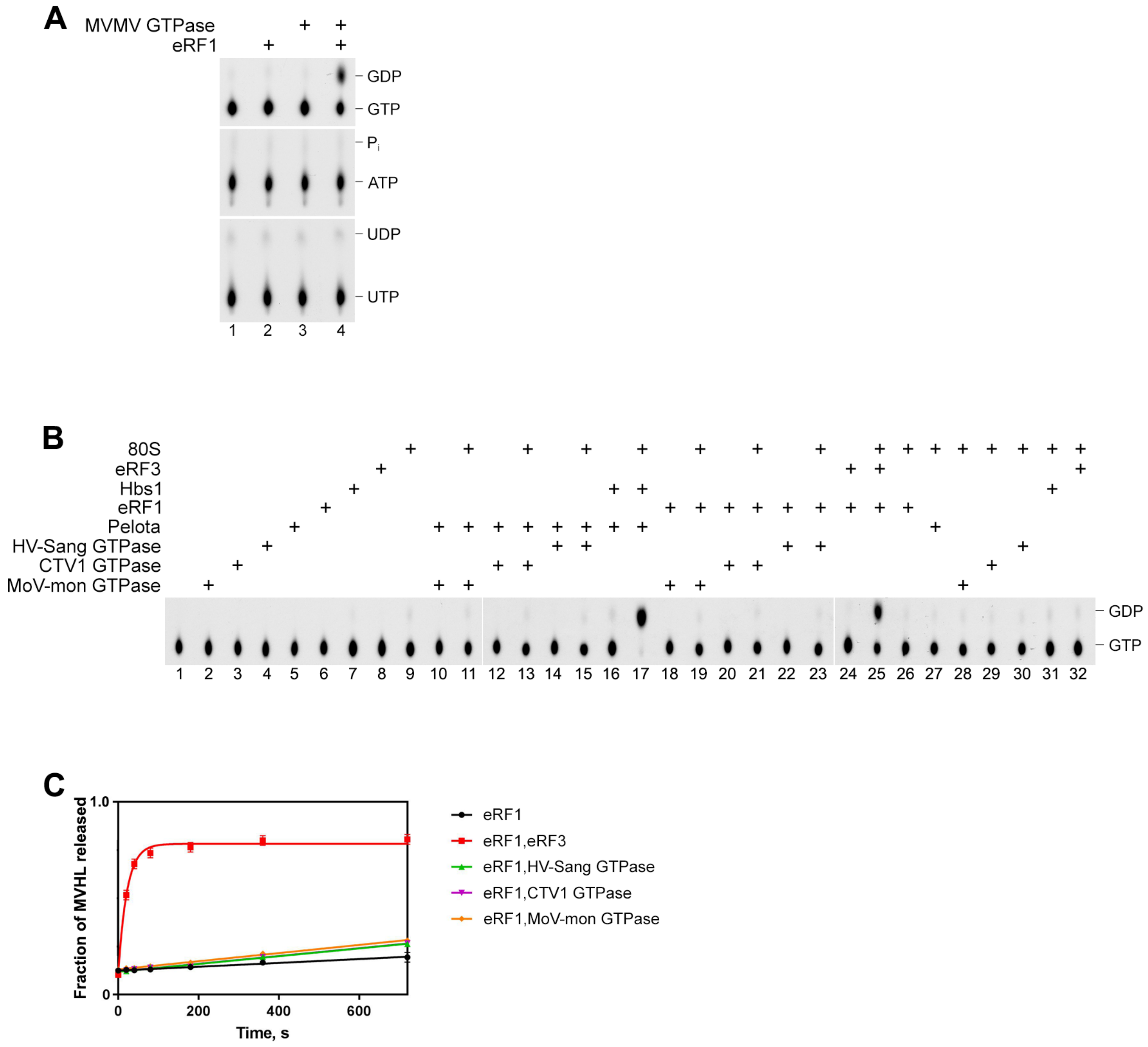


**Figure S2. The activities of MVMV, LV, CTV1, HV-Sang and MoV-mon GTPases in termination, related to Figure 3.**

(A) Hydrolysis of $[\alpha\text{-}^{32}P]$GTP, $[\alpha\text{-}^{32}P]$ATP and $[\gamma\text{-}^{32}P]$UTP by the MVMV GTPase in the presence/absence of eRF1, assayed by TLC followed by autoradiography. (B) TLC analysis of $[\alpha\text{-}^{32}P]$GTP hydrolysis by MoV-mon, CTV1 and HV-Sang GTPase in the presence/absence of different combinations of 80S ribosomes, eRF1 and Pelota, as indicated. In control experiments, $[\alpha\text{-}^{32}P]$GTP is hydrolyzed by eRF3 in the presence of 80S ribosomes and eRF1, and by Hbs1 in the presence of 80S ribosomes and Pelota. (C) Kinetics of $[^{35}S]$MVHL tetrapeptide release by human *wt* eRF1 alone (black), or in the presence of human eRF3 (red), HV-Sang GTPase (green), CTV1 GTPase (magenta), or MoV-mon GTPase (orange). The concentrations of pre-TCs, eRF1 and eRF3/HV-Sang/CTV1/MoV-mon GTPases were 2 nM, 10 nM and 20 nM, respectively. Each time point represents the average of three independent experiments. Error bars represent SD.

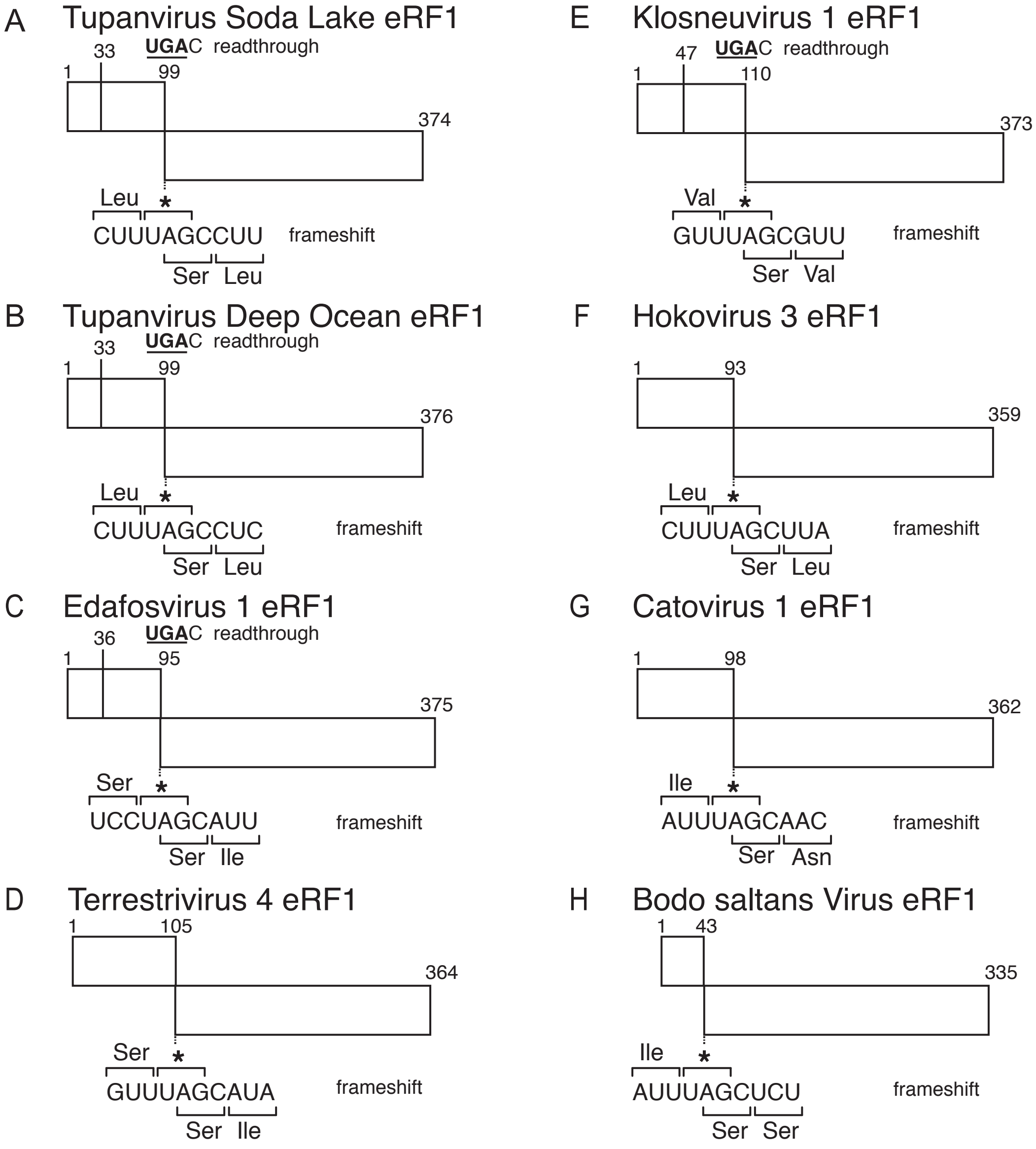


**Figure S3. Schematic representations of eRF1 coding regions in recently described members of *Mimiviridae* (6, 7, 10, 11), showing the potential for synthesis of full-length polypeptides by stop-codon readthrough and frame-shifting.** Coding regions are from (A) Tupanvirus Soda Lake (KY523104.1), corresponding to nt.1395607-1396732, which include the coding region AUL78710.1 and (B) Tupanvirus Deep Ocean (MF405918.1), corresponding to nt. 1506807-1507938, which include the coding region AUL80095.1 (11), (C) Edafosvirus 1 (MK072066.1), complementary to nt. 71863-70735, which include the coding regions AYV77739.1 and AYV77740.1 and (D) Terrestrivirus 4 (MK071982.1), corresponding to nt. 16828- 17923, which include the coding regions AYV75969.1 and AYV75970.1 (7), (E) Klosneuvirus 1 (KY684108.1), complementary to nt. 316634- 315042, which include the coding region ARF11430.1, (F) Hokovirus 3 (KY684105.1), complementary to nt. 378104 - 377024, which include the coding regions ARF10995.1 and ARF10994.1, and (G) Catovirus 1 (KY684083.1), corresponding to nt. 986668- 987757, which includes the coding regions ARF08894.1 and ARF08895.1 (10), and (H) Bodo saltans virus strain NG1 (MF782455), corresponding to nt. 589232- 590258, which includes the coding regions ATZ80562.1 and ATZ80563.1 (6). Stop codon read-through in these and other members of *Mimiviridae* would occur at the conserved sequence **UGA**C (stop codon bold and underlined), which supports the highest level of stop codon suppression in eukaryotes (72), whereas the frame-shifting consensus sequence CUU**UAG**C in the viral sequences resembles the CUU **UGA** shift site/stop codon cassette that promotes +1 frame-shifting in the bacterial polypeptide chain release factor 2 (73).

| Family and lineage/ Sub-clade | Virus | GTPase | eRF1 | Arginyl-tRNA synthetase | Aspartyl/ Asparaginyl tRNA synthetase | Cysteinyl-tRNA synthetase | Isoleucyl-tRNA synthetase | Methionyl-tRNA synthetase | Tryptophanyl tRNA synthetase | Tyrosyl-tRNA synthetase | tRNAs |
|---|---|---|---|---|---|---|---|---|---|---|---|
| ***Marseilleviridae*** | | | | | | | | | | | |
| Subclade A | Marseillevirus | ADB03944 | ADB03912 | x | x | x | x | x | x | x | x |
| | Cannes 8 Virus | AGV01518 | AGV01484 | x | x | x | x | x | x | x | x |
| | Melbournevirus | AIT54760.1 | AIT54729 | x | x | x | x | x | x | x | x |
| Subclade B | Lausannevirus | AEA06904 | AEA07018 | x | x | x | x | x | x | x | x |
| | Port-Miou virus | ALH06743 | ALH06844 | x | x | x | x | x | x | x | x |
| Subclade C | Tunisvirus fontaine 2 | AHC54765 | AHC54787 | x | x | x | x | x | x | x | x |
| | Insectomime virus | AHA46163 | AHA46142 | x | x | x | x | x | x | x | x |
| Subclade D | Brazilian marseillevirus | AMQ10670 | AMQ10707 | x | x | x | x | x | x | x | x |
| ***Mimiviridae*** | | | | | | | | | | | |
| Lineage A | *A. polyphaga* mimivirus | ADO18750 | AKI79513 (Frameshift & Readthrough) | ADO18783 | x | ADO18016 | x | ADO18762 | x | ADO17990 Readthrough | 3 Leu 1His 1 Cys 1 Trp |
| | Hirudovirus | AHA45220 | AHA45106 AHA45107 (Frameshift & Readthrough) | AHA45176 | x | AHA45707 | x | AHA45707 | x | AHA45751 Readthrough | 3 Leu 1His 1 Cys 1 Trp |
| Lineage B | *A. polyphaga* moumouvirus | ADX97534 | JX962719 nt 205481-206699 (Frameshift & Readthrough) | AGC02245-AGC02248 Frameshift & Readthrough | AGC02177 | ADX97544 Readthrough | AGC01825 | AGC02204 Readthrough | x | AGC02320 (Frameshift) | 1 Leu 1His 1 Cys |
| | Moumouvirus monve | AEX62488 | AEX63065 AEX63067 (Frameshift & Readthrough) | AEX62415 AEX62416 Readthrough | AEX62498 | AEX62409 | AEX62962 AEX62963 Frameshift | AEX62460 | x | AEX62320 (Frameshift) | 1 His 1 Cys |
| Lineage C | Megavirus chiliensis | AEQ32879 | AEQ32494 (Frameshift & Readthrough) | AEQ32742 | AEQ33079 | AEQ33342 | AEQ33448 | AEQ33119 | AEQ32410 | AEQ32875 | 2 Leu 1 Trp |
| | Megavirus Iba | AGD92699 | AGD92187 AGD92188 ((Frameshift & Readthrough) | AGD92756 | AGD92690 | AGD92759 | AGD92264 | AGD92718 | AGD92799 | AGD92873 | 2 Leu 1His 1 Cys 1 Trp |
| | Megavirus Terra1 | ADX97533 | KF527229 (Frameshift & Readthrough) | ADX97536 (Frameshift) | KF527229 (nt. 1221260-1219843) (Frameshift) | ADX97543 | KF527229 (nt. 433073-429774) | ADX97538 | KF527229 (nt. 804593-805789) | ADX97540 | 2 Leu 1 Trp |

Table S1. Selected components of the translation apparatus encoded by representative members of Marseilleviridae and Mimiviridae (GenBank accession codes correspond to coding regions or fragment(s) thereof.
